# Can horses read emotional cues from human faces? Re-analysis of Smith *et al.* (2016)

**DOI:** 10.1098/rsbl.2016.0201

**Published:** 2016-09

**Authors:** Tim Schmoll

**Affiliations:** Evolutionary Biology, Bielefeld University, Morgenbreede 45, 33615 Bielefeld, Germany

In a recent *Biology Letters* article, Smith and co-workers reported that horses displayed a left-gaze bias and a quicker increase in heart rate (HR) when presented with stimulus photographs depicting angry versus happy human faces [1]. Here, I flag up a number of concerns, which collectively suggest the main conclusions of this contribution are not supported by the currently available data.

In their first major analysis, the authors established that the probability of left-gaze first looks was significantly different from chance when presenting an angry stimulus, but not significantly different from chance when presenting a happy stimulus (Fig. 2a in [1]). In a related second major analysis, they established that their laterality index was significantly different from zero when presenting an angry stimulus, but not significantly different from zero when presenting a happy stimulus (Fig. 2b in [1]). In the conclusions, these results are interpreted to show “… first evidence of horses' abilities to spontaneously discriminate … between positive and negative human facial expressions …”. This inference is, however, not supported by the abovementioned analyses, which represent inappropriate comparisons of differences in nominal significance within instead of between treatment groups [2]. The presence of significant differences from chance/zero in one group (angry) and absence of significant differences from chance/zero in the other (happy) *cannot* demonstrate that the *responses differed according to the stimulus type*, i.e. that the differences between treatment levels were significantly different from zero: only direct comparisons of treatment levels can demonstrate the claimed discrimination ability [2]. Importantly, any inference based on nominal significance when analysing behavioural responses only within groups is fundamentally flawed here as the observed deviation from chance in the behavioural responses following presentation of *angry* faces (see above) cannot be distinguished from responses following presentation of *any kind* of faces or from the possibility that the test set-up was perceived stressful by subjects in general (see below) or from the possibility that the lateralized positioning of the experimenter (always on the horses' left shoulders) has biased the behavioural response (see below).

To test whether lateralized looking behaviours actually differed according to stimulus type, I re-analysed first looks using a generalized linear mixed model with binomial errors and logit link. I included *Emotion* (angry/happy) as fixed effect and horse identity as random effect to account for multiple measurements on the same subject. There was no significant difference between stimulus types in the probability to first look with the left eye towards stimuli (likelihood ratio test: *χ*^2^ = 0.59, d.f. = 1, *p* = 0.44). There was, however, some evidence for a systematic left-gaze bias in first looks independent of stimulus type (difference of the intercept, i.e. the overall mean probability to first left-gaze, from zero on the logit scale, i.e. from chance: *z* = –2.41, *p* = 0.02). Both results are easily reconciled with the graphical representation of the data in Fig. 2a in [1].

Likewise, I re-analysed the laterality index using a linear mixed effects model with identical predictor variables as above. There was no significant difference in the laterality index between stimulus types (*t*_28_ = 1.51, *p* = 0.14). There was, however, strong evidence that horses generally spent more time adopting a left-gaze posture during the test independent of stimulus type (difference of the intercept, i.e. the overall mean laterality index, from zero: *t*_56_ = 3.53, *p* = 0.0008). Both results are easily reconciled with Fig. 2b in [1]. In fact, the first part of the mixed model analysis for the laterality index is largely equivalent to a paired *t*-test, results of which had actually been provided in the legend of Fig. 2 in [1]: “There were no significant differences in looking durations when the valences were directly compared, *t*_27_ = –1.49, *p* = 0.15 (paired-samples *t*-test, two-tailed)”. The authors appear to have failed to recognize this test as being the more appropriate for their research question as its outcome was neither otherwise mentioned in the results section nor taken up in the discussion.

The systematic left-gaze bias could result from the fact that the test situation was perceived stressful by subjects independent of stimulus type. This interpretation is compatible with the reported significant correlations between time spent looking left, time spent avoiding and HR difference test versus baseline [1]. Alternatively, such a bias could also represent an artefact of the test set-up given that experimenter 1 always stood on the horses' left shoulders during trials [1]. To firmly exclude this when studying lateralized behaviours, experimenters should preferentially be positioned on either side of the test subjects following a balanced design.

In a third major analysis, the paper addressed the latency until horses reached maximum HR during the trial subsequent to stimuli presentation. Why this measure is problematic is best exemplified by the latency recorded for subject *Jack* when presented with an angry stimulus during experimental round 2: this ‘latency’ was zero seconds [3], which cannot possibly reflect a response to any stimulus presented. Moreover, a ‘latency’ of zero essentially means that *Jack* decreased HR (i.e. calmed down) upon presentation of an angry stimulus. Two more ‘latencies’ in the angry stimulus group amounted to just 0.4 s each and are equally problematic: given an overall mean HR of 39.4 ± 9.3 (s.d.) per minute [3], these time intervals can also not possibly reflect real responses to the stimulus. However, the analytical approach adopted by the authors takes these values—modelled erroneously as latencies in response to the stimulus—to represent the most extreme stress reactions possible. This unjustifiably biases the test outcome towards subjects appearing more stressed when seeing angry faces. As evidenced by the occurrence of values with no sensible biological interpretation, this variable cannot be considered well suited to measure meaningful responses to the stimuli. Taking into account that no treatment effects were demonstrated for the biologically more sensible measures *HR change between baseline and test*, *absolute maximum HR* or *recovery time*, I conclude there is at present no evidence for any biologically relevant effects of human facial expressions of emotions on HR in horses.

## Supplementary Material

Supplementary methods

## Supplementary Material

R script of re-analysis

## Supplementary Material

Raw Data Behaviour

## Supplementary Material

Raw Data Heart Rate

## Data Accessibility

Details of the statistical methods including the R code used are documented in the electronic supplementary material.
