# Supplementary material for: Can horses read emotional cues from human faces? Re-analysis of Smith et al. (2016)
Source: Biol Lett. 2016 Sep;12(9):20160201. doi: 10.1098/rsbl.2016.0201 (PMC5046913; doi:10.1098/rsbl.2016.0201)
Supplement: Supplementary methods [file rsbl20160201supp1.docx]

**Electronic Supplementary Material for article “Can horses read emotional cues from human faces? Re-analysis of Smith et al. *Functionally relevant responses to human facial expressions of emotion in the domestic horse (Equus caballus),* Biology Letters 12, 20150907”.**

**Supplementary methods**

**Data processing**

I used the data deposited for this article at Dryad for my re-analysis [1]. In both data sheets (*Behaviour*, *Heart Rate*) I replaced space characters with dots and saved each data sheet as a tab-delimited .txt file for input into R.

**Statistical analysis**

I used R 3.3.0 [2] and generalized linear mixed effects models (GLMM) with binomial error distribution and logit link function (R function *glmer* from package *lme4* [3]) and linear mixed effects models (LME, R function *lmer* from package *lme4* [3]) to test for effects of stimulus type on horse behaviour. I included subject identity as random effect to account for the dependency of measurements from the same subject. For significance testing I used likelihood ratio tests in the case of GLMMs and t-tests with Satterthwaite approximation to degrees of freedom as implemented in the package *lmerTest* [4] in the case of LMEs. R code documenting the re-analysis can be found in the electronic supplementary file *Smith et al. 2016 Re-analysis Schmoll.r*.

**References**

[1] Smith, A.V., Proops, L., Grounds, K., Wathan, J. & McComb, K. 2016 Data from: Functionally relevant responses to human facial expressions of emotion in the domestic horse (*Equus caballus*). Dryad Digital Repository. <http://dx.doi.org/10.5061/dryad.2m6t6>

[2] R Core Team 2016 R: A language and environment for statistical computing. R Foundation for Statistical Computing, Vienna, Austria. URL. <https://www.R-project.org/>.

[3] Bates, D., Maechler, M., Bolker, B. & Walker, S. 2015. Fitting Linear Mixed-Effects Models Using lme4. *J Stat Softw* **67**, 1-48. doi:10.18637/jss.v067.i01.

[4] Kuznetsova, A., Bruun Brockhoff, P. & Christensen, R.H.B. 2016 lmerTest: Tests in Linear Mixed Effects Models. R package version 2.0-30. <https://CRAN.R-project.org/package=lmerTest>
